# *MhYTP1* and *MhYTP2* from Apple Confer Tolerance to Multiple Abiotic Stresses in *Arabidopsis thaliana*

**DOI:** 10.3389/fpls.2017.01367

**Published:** 2017-08-04

**Authors:** Na Wang, Tianli Guo, Ping Wang, Xun Sun, Yun Shao, Xin Jia, Bowen Liang, Xiaoqing Gong, Fengwang Ma

**Affiliations:** State Key Laboratory of Crop Stress Biology for Arid Areas, College of Horticulture, Northwest A&F University Yangling, China

**Keywords:** YTH domain, promoter, phytohormone, stress, *Malus*

## Abstract

The first YTH domain-containing RNA binding protein (YTP) was found in rat, where it was related to oxidative stress. Unlike characterizations in yeast and animals, functions of plant YTPs are less clear. *Malus hupehensis* (Pamp.) Rehd. YTP1 and YTP2 (MhYTP1 and MhYTP2) are known to be active in leaf senescence and fruit ripening. However, no research has been published about their roles in stress responses. Here, we investigate the stress-related functions of *MhYTP1* and *MhYTP2* in *Arabidopsis thaliana*. Both of the two genes participated in salicylic acid (SA), jasmonic acid (JA), and abscisic acid (ABA) signaling and play roles in plant responses to oxidative stress, chilling, high temperature, high salinity, and mannitol induced physiological drought stress. Moreover, *MhYTP1* plays leading roles in SA and ABA signaling, and *MhYTP2* plays leading roles in JA signaling and oxidative stress responses. These results will fill a gap in our knowledge about plant YTPs and stress responses and provide a foundation for future attempts to improve stress tolerance in apple.

## Introduction

Phytohormones such as, abscisic acid (ABA), jasmonic acid (JA), and salicylic acid (SA) play vital roles in stress responses (Reje et al., 2014). For example, ABA is active when plants are exposed to heavy metals, drought, extremes in temperature, high salinity, or radiation. It also functions in various developmental processes, including seed germination and dormancy, and the closure of stomata (Vishwakarma et al., 2017). Both JA and SA are involved in responses to biotic stresses. The JA-signaling pathway is critical to the development of resistance to necrotrophic pathogens that derive nutrients from dead or dying cells, while the SA-signaling pathway is mainly activated against biotrophic pathogens that obtain nutrients from living host tissues (Glazebrook, 2005). In plants resistant to pathogen infections, the JA- and SA-defense pathways generally interact antagonistically (Verhage et al., 2010).

When plants are stressed, their hormone signaling is altered, which then changes the expression of responsive genes. In the gene regulation network, RNA binding proteins (RBPs) participate at the transcriptional and post-transcriptional levels, which is a powerful strategy of gene expression regulation. The most common post-transcriptional modifications are alternative splicing and polyadenylation, which change RNA sequences, protein functioning, and the availability of RNA-binding sites (Filichkin et al., 2010; Di Giammartino et al., 2011). The RBPs bind with target RNAs via their RNA-binding domains. To date, approximately 40 types of RNA-binding domains have been identified from various RBPs (Kishore et al., 2010). The novel YTH (for YT521-B homology) domain and YTH domain-containing RNA binding proteins (YTPs) have been described in yeast, animals (including humans; *Homo sapiens*), and plants (Stoilov et al., 2002).

The first identified YTP was YT521, found in the astrocytes of rat (*Rattus norvegicus*). The mRNA level of *YT521* can be depressed under oxygen-deficient conditions but recovered after reoxygenation. Proteins that interact with YT521 are also RNA-binding proteins, e.g., RA301, SC35, and SF2, which indicates that *YT521* is involved in post-transcriptional regulation (Imai et al., 1998). Another YTP gene, *YT521-B*, cloned from the brain library of rat, can alter the selection of alternative splicing sites in a concentration-dependent manner (Hartmann et al., 1999). *Mmi1*, a YTP in the model system *Schizosaccharomyces pombe* (fission yeast), is important in the molecular regulation of meiosis and controls sexual differentiation in fission yeast (Harigaya et al., 2006; Chen et al., 2011; Hiriart et al., 2012). However, meiosis-specific mRNAs are eliminated by binding to *Mmi1* during the vegetative growth phase (Harigaya et al., 2006). Four YTPs occur in humans, and various *YTHDF2* loci are associated with human longevity (Cardelli et al., 2006). Recently, researchers are focusing on functions of YTHDF2 and YTHDF3 in binding with the methylated N6 position of selected internal adenines in mRNAs and non-coding RNAs, which affect the translation status and lifetime of mRNA (Li F. et al., 2014; Zhu et al., 2014; Shi et al., 2017). These studies with yeast and animal YTPs have primarily focused on interactions between YTPs and target RNAs, and have not examined their roles in stress responses.

However, the information about plant YTPs are limited in contrast with researches on animals and yeast. The first plant YTP—cleavage and polyadenylation specificity factor 30 (CPSF30)—was discovered in *Arabidopsis*, where it participates in defense and oxidative-stress responses, as well as cellular signaling (Addepalli and Hunt, 2007; Chakrabarti and Hunt, 2015). Li D. et al. (2014) have identified YTP family members in *Arabidopsis* and analyzed their functions in abiotic-stress responses. Fifteen members of the *Malus* YTP family have been reported and their expression has been investigated via quantitative real-time PCR (qRT-PCR) experiments during leaf senescence and under various abiotic-stress conditions (Wang et al., 2014). Although *Malus hupehensis* (Pamp.) Rehd. YTP1 and YTP2 (MhYTP1 and MhYTP2) have functions in leaf senescence and fruit ripening (Wang et al., 2017), their roles in stressed plants have not been confirmed.

Here, we investigated the responses of *MhYTP1* and *MhYTP2* to various environmental stimuli in *Arabidopsis thaliana*, based on their promoter *cis*-elements. After fusion with the β-glucuronidase (GUS) gene, their promoters were transferred into *Arabidopsis* plants. We also treated plants that over-expressed *MhYTP1* or *MhYTP2* and tested the responses of those transgenics to SA, methyl jasmonate (MeJA), ABA, methyl viologen (MV), exposure to 4 or 40°C, salt (NaCl), or mannitol.

## Materials and methods

### Cloning and sequence analysis of genes and promoters

We previously determined the sequences of *MhYTP1* (MDP0000124048) and *MhYTP2* (MDP0000488588) from the Genome Database for Rosaceae (GDR; Wang et al., 2017). For promoter cloning, we used a CTAB-based method (Porebski et al., 1997) to isolate genomic DNA from young leaves of 1-year-old *M. hupehensis* seedlings. The promoter regions were cloned by PCR, based on the region upstream of *MhYTP1* (GDR: contig MDC008177.673; NCBI: MF370872) or *MhYTP2* (GDR: contig MDC011033.286; NCBI: MF370873). All primers are listed in Supplementary Table 1. The functions of *cis*-acting elements in those promoters were predicted from the PlantCARE database (Rombauts et al., 1999; Postel et al., 2002).

### Production of transgenic *Arabidopsis thaliana* plants

To construct our GUS expression vectors, we inserted the promoter regions of *MhYTP1* and *MhYTP2* into 433 vectors (Gateway; *att*R1-*att*R2-GUS-T_NOS_). For construction of the overexpression (OE) vectors, the open reading frame of *MhYTP1* or *MhYPT2* was inserted into the pCambia2300 vector. These vectors were then transferred to *Agrobacterium tumefaciens*. Transformation and selection of transgenic *Arabidopsis* plants were performed by the floral-dip procedure (Zhang et al., 2006). Before being spread on wet soil in pots, seeds of the wild type (WT) *A. thaliana* (Columbia, or Col-0, ecotype) were suspended in re-distilled water and kept in the dark for 3 days at 4°C. After the seeded pots were placed in a growth chamber under short-day conditions (8-h photoperiod) for 3–4 weeks, they were moved to long-day conditions (16-h photoperiod) to induce flowering.

A positive single *Agrobacterium* colony was inoculated into a liquid YEB medium containing appropriate antibiotics and incubated at 28°C. After the OD_600_ reached 1.5–2.0, *Agrobacterium* cells were collected by centrifugation at 4,000 g for 10 min and re-suspended in a fresh 5% (w:v) sucrose solution with 0.02% (v:v) Silwet L-77. The *Arabidopsis* plants were inverted and their aerial parts dipped into this cell suspension for 10 s. Afterward, those plants were kept under darkness for 16–24 h before being returned to the growth chamber.

To select transgenic plants, we sterilized T_0_ seeds with 70% ethanol for 1 min and 5% bleach for 10 min. After being rinsed three times with sterile water, the seeds were re-suspended in 0.05% agarose and spread on an MS selection agar medium supplemented with kanamycin. Plates were held at 4°C for 3 days and then transferred to 22°C under short-day conditions. Transgenic plants were distinguishable after 7–10 days. Preliminarily confirmed T_0_ transgenic plants were transferred to fresh selection plates and grown for another 1–2 weeks. Afterward, fully verified plantlets were transferred to soil in pots, and then moved to a growth chamber for seed development. Homozygous T_3_ seeds were produced by T_2_ transgenic plants that had survived on the selection media. Homozygous lines were further confirmed by qRT-PCR.

### Quantitative real-time PCR

Total RNA from snap-frozen *Arabidopsis* seedlings was extracted by a modified CTAB method (Gambino et al., 2008). The cDNA was reversed-transcribed from total RNA using a PrimeScript® RT reagent Kit with gDNA Eraser (Perfect Real Time; Takara). Quantitative real-time PCR was performed according to protocols for SYBR® Premix Ex Taq™ II (Tli RNaseH Plus; Takara) and the Real Time PCR System machine (Bio-Rad iQ™ 5; Bio-Rad). The gene-specific primers are shown in Supplementary Table 1. Expression levels were calculated relative to the expression of *AtTublin8* mRNA.

### Stress treatments

For testing the responses of *Arabidopsis* to environmental inductions, seeds of the WT and homozygous T_3_ transgenic plants were surface-sterilized and germinated on a standard MS medium. After 7 days, seedlings of all lines were transferred to either a standard MS medium or one that was supplemented with SA (10 μM), MeJA (5 μM), ABA (2 μM), MV (1 μM), NaCl (200 mM), or mannitol (200 mM). After 7 days of stress treatment, *GUS* expression was investigated in both WT and transgenic plants. Fresh weights and root lengths were recorded from WT and OE plants of each treatment.

The response to chilling (4°C) or heat (40°C) was examined using 14-day-old seedlings grown for 4 days on standard MS media, and was compared with the performance of plants under normal (control) conditions, i.e., 25°C. Again, *GUS* expression was monitored for both WT plants and those transformed with *MhYTP1* or *MhYTP2* promoters. Relative electrolyte leakage (REL) and the concentration of total chlorophyll were calculated for leaf samples from WT and OE plants under normal and treatment conditions. The REL was measured with a conductivity meter (Leci; DDS-307). Chlorophyll was extracted with 80% acetone and concentrations were determined spectrophotometrically according to the methods of Lichtenthaler and Wellburn (1983).

### Statistical analysis

Data were subjected to one-way ANOVA, and mean differences were assessed by Duncan analyses (statistically significant at *P* < 0.05).

## Results

### *MhYTP1* and *MhYTP2* participate in SA, JA, and ABA signaling

**Figure 1:**
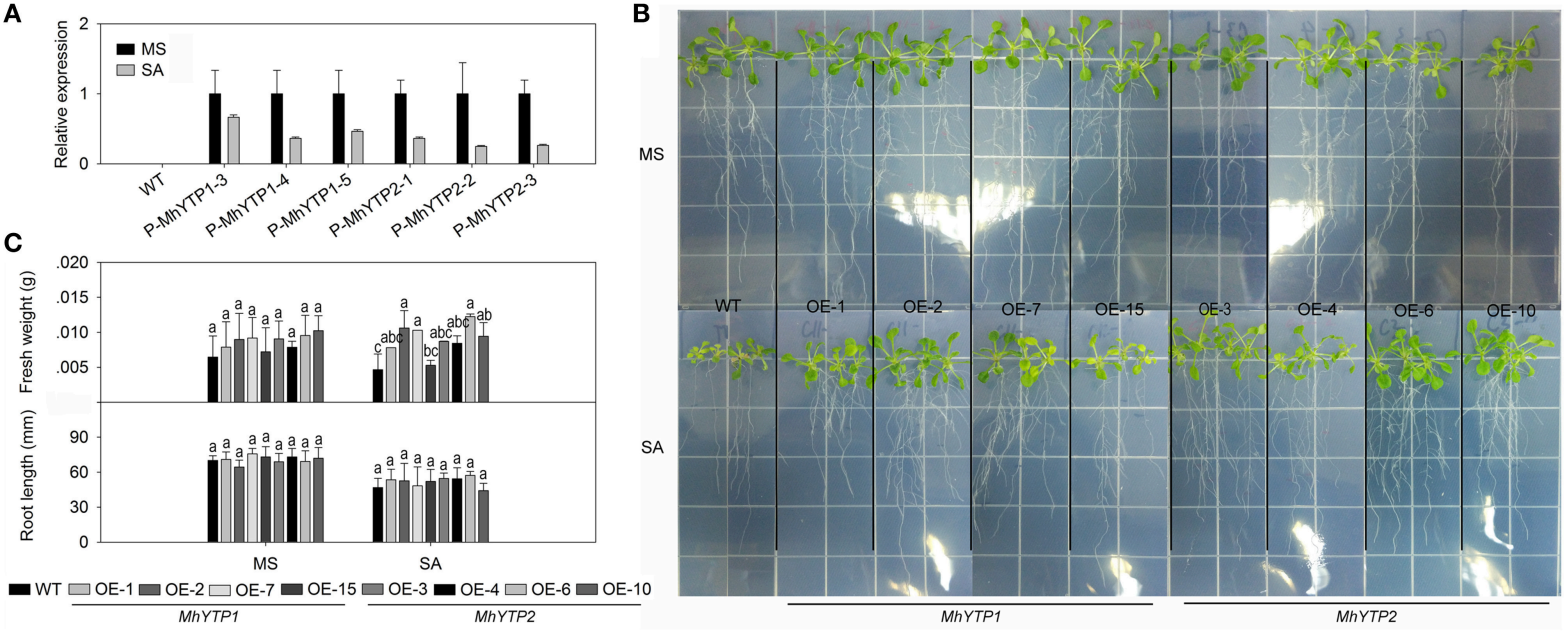
**(A)** Relative expression levels of *GUS* in WT and transgenic plants under normal conditions or salicylic acid (SA) treatment. Data are means ± SD for 5 replicates (at least 20 seedlings each). **(B)** Phenotype comparisons between WT and *MhYTP1-*overexpression plants, and between WT and *MhYTP2*-overexpression plants, in standard MS medium and under SA treatment. **(C)** Fresh weights and root lengths of OE and WT *Arabidopsis* plants grown in standard MS medium or MS medium supplemented with 10 μM SA. Data are means of 15 replicates ± SD. Values not followed by same letter are significantly different according to Duncan's multiple range test (*P* < 0.05).

Because the *MhYTP1* promoter region contains a SA signaling-responsive *cis*-element (TCA-Element), we first investigated whether our two promoters could be induced by exogenously applied SA (Supplementary Figure 1). After they were fused with the GUS reporter gene in the expression vector, the constructs were transferred to *Arabidopsis* plants (Supplementary Figure 2). Our results indicated that SA treatment led to a decline in *GUS* expression levels in those transgenics (Figure 1A). We also over-expressed those YTP genes separately in *Arabidopsis* and obtained Lines OE-1, OE-2, OE-7, and OE-15 for *MhYTP1*; and Lines OE-3, OE-4, OE-6, and OE-10 for *MhYTP2* (Supplementary Figure 3). After growing on either normal MS or an MS medium supplemented with 10 μM SA for 7 days, values for fresh weights were higher for all OE plants than for the WT. This indicated that the latter genotype was more sensitive to SA (Figures 1B,C). In contrast, we found no significant differences in root lengths between WT and OE plants.

**Figure 2:**
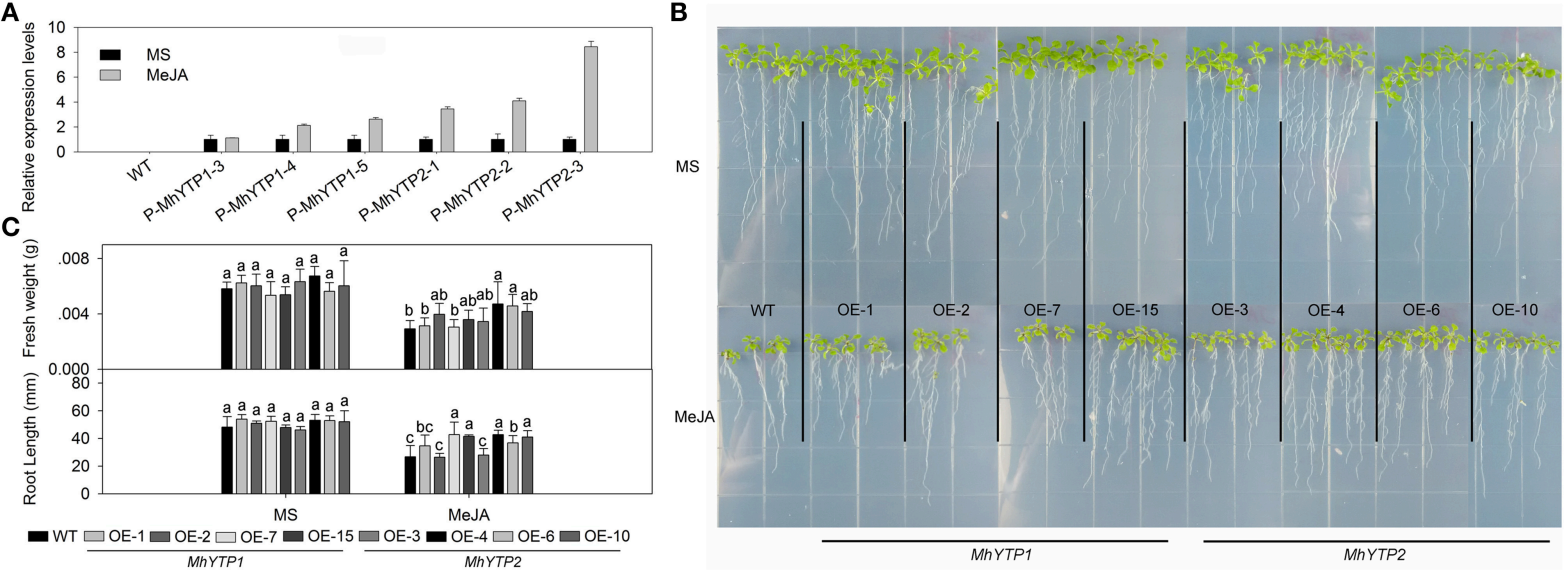
**(A)** Relative expression levels of *GUS* in WT and transgenic plants under normal conditions or methyl jasmonate (MeJA) treatment. Data are means ± SD for 5 replicates (at least 20 seedlings each). **(B)** Phenotype comparisons between WT and *MhYTP1-*overexpression plants, and between WT and *MhYTP2*- overexpression plants, in standard MS medium and under MeJA treatment. **(C)** Fresh weights and root lengths of OE and WT *Arabidopsis* plants grown in standard MS medium or MS medium supplemented with 5 μM MeJA. Data are means of 15 replicates ± SD. Values not followed by same letter are significantly different according to Duncan's multiple range test (*P* < 0.05).

Both JA signaling and SA signaling participate in plant defenses against pathogen infection, and their pathways interact with each other. We noted that *GUS* expression was induced by MeJA treatment, especially in plants transformed with the *MhYTP2* promoter region (Figure 2A). Transcript levels of *GUS* were up to nine-fold higher in the treated transgenics than in the untreated plants. Separately, OE plants were generally more resistant to exogenously applied MeJA, as evidenced by their heavier fresh weights and longer root lengths when compared with the WT (Figures 2B,C). However, fresh weight of *MhYTP1* overexpression line OE-1 and OE-7 are the same with that of WT; root lengths of WT, *MhYTP1* overexpression line OE-2, *MhYTP2* overexpression line OE-3 have no significant differences.

**Figure 3:**
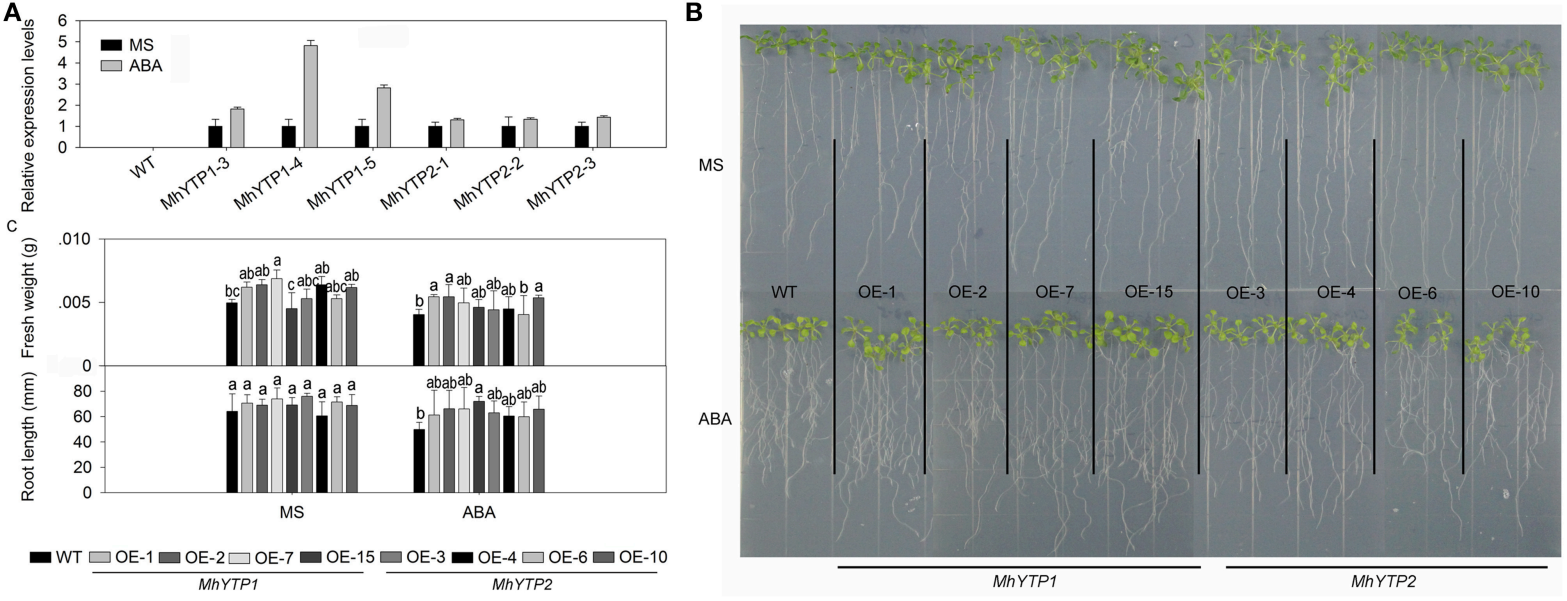
**(A)** Relative expression levels of *GUS* in WT and transgenic plants under normal conditions or abscisic acid (ABA) treatment. Data are means ± SD for 5 replicates (at least 20 seedlings each). **(B)** Phenotype comparisons between WT and *MhYTP1-*overexpression plants, and between WT and *MhYTP2*-overexpression plants, in standard MS medium and under ABA treatment. **(C)** Fresh weights and root lengths of OE and WT *Arabidopsis* plants grown in standard MS medium or MS medium supplemented with 2 μM ABA. Data are means of 15 replicates ± SD. Values not followed by same letter are significantly different according to Duncan's multiple range test (*P* < 0.05).

The ABA-treated plants transformed with the *MhYTP1* promoter also show induced *GUS* expression, whereas the level of expression in plants transformed with the *MhYTP2* promoter did not differ significantly from their corresponding untreated plants (Figure 3A). After 7 days of growth on standard MS or the medium supplemented with 2 μM ABA, values for fresh weights and root lengths were higher for both *MhYTP1-*OE and *MhYTP2-*OE plants than for the WT (Figures 3B,C), indicating that the non-transformed plants were more sensitive to ABA.

### *MhYTP1* and *MhYTP2* can be induced by MV, and overexpression plants are more resistant to MV treatment

**Figure 4:**
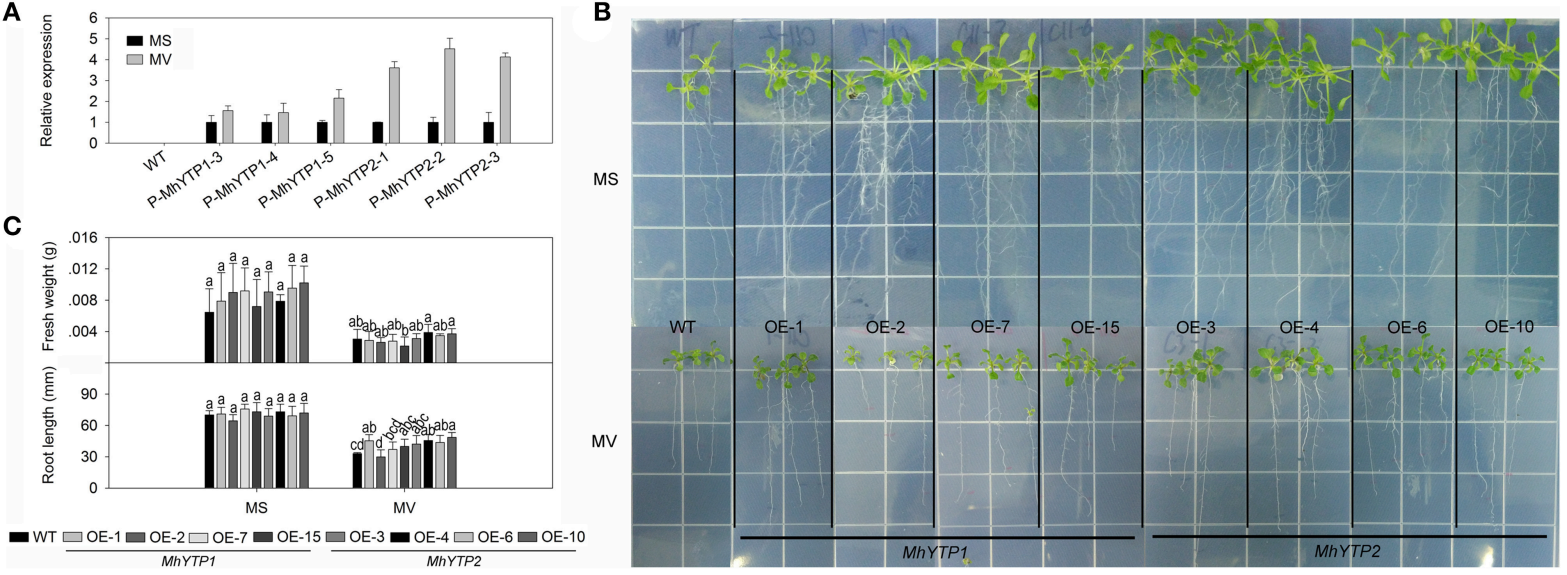
**(A)** Relative expression levels of *GUS* in WT and transgenic plants under normal conditions or methyl viologen (MV) treatment. Data are means ± SD for 5 replicate samples (at least 20 seedlings each). **(B)** Phenotype comparisons between WT and *MhYTP1-*overexpression plants, and between WT and *MhYTP2*-overexpression plants, in standard MS medium and under MV treatment. **(C)** Fresh weights and root lengths of OE and WT *Arabidopsis* plants grown in standard MS medium or MS medium supplemented with 1 μM MV. Data are means of 15 replicates ± SD. Values not followed by same letter are significantly different according to Duncan's multiple range test (*P* < 0.05).

The presence of one ARE (anaerobic induction element) in the *MhYTP1* promoter region as well as ARE and GC-motif (anoxic specific induction) elements in the *MhYTP2* promoter region demonstrated their roles in the hypoxia-stress response (Supplementary Figure 1). Hypoxic conditions can cause oxidative stress in plants due to the generation of reactive oxygen species (ROS). Here, MV treatment resulted in greater *GUS* expression in plants carrying either the *MhYTP1* or *MhYTP2* promoter, and the response was especially strong when *GUS* was fused with the latter (Figure 4A).

After 7 days of growth on normal MS or MS media containing 1 μM MV, the roots were generally longer from OE plants than from the WT (Figures 4B,C), indicating that the latter was more sensitive to this stress treatment. The exception was for the roots of *MhYTP1-*OE 2, which were shorter than those of either *MhYTP2-*OE plants or the WT. *MhYTP2* overexpression plants grow better than *MhYTP1* overexpression and WT plant. A comparison of fresh weight values revealed no significant difference among genotypes, although *MhYTP1-*OE 15 was a bit lighter and *MhYTP2-*OE 4 and 10 were slightly heavier than the WT plants.

### *MhYTP1* and *MhYTP2* play roles in chilling and heat stress responses

**Figure 5:**
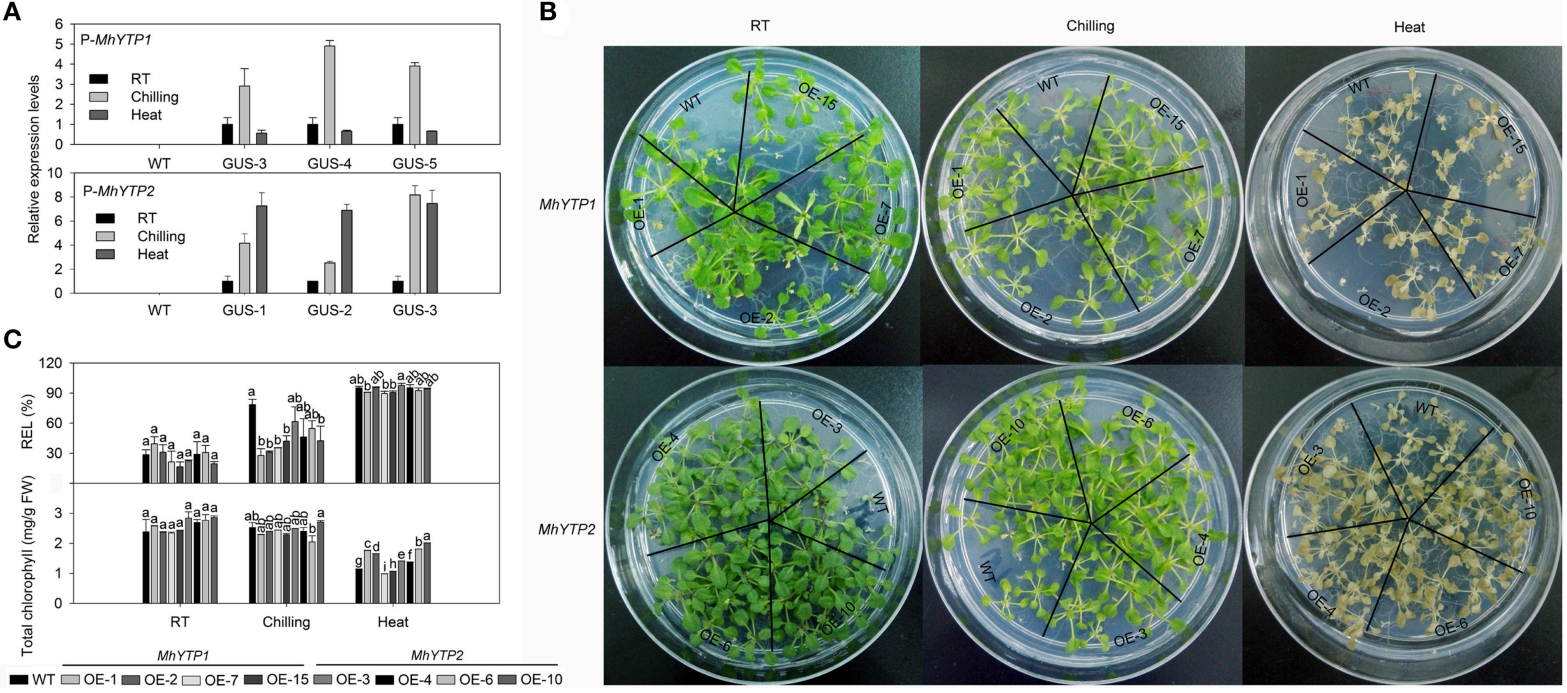
**(A)** Relative expression levels of *GUS* in WT and transgenic plants under normal (25°C), chilling (4°C), or heat-stress (40°C) conditions. Data are means ± SD for 5 replicates (at least 20 seedlings each). **(B)** Phenotype comparisons between WT and *MhYTP1-*overexpression plants, and between WT and *MhYTP2*-overexpression plants under normal, chilling, or heat treatment. **(C)** Relative electrolytic leakage (REL) in leaves and concentrations of total chlorophyll in OE and WT *Arabidopsis* plants grown under normal, chilling, or heat treatment. Data are means of 5 replicates ± SD. Values not followed by same letter are significantly different according to Duncan's multiple range test (*P* < 0.05).

A HSE (heat stress response element) was found in the promoter region for *MhYP2* but not for *MhYTP1* (Supplementary Figure 1). Consistent with this, *GUS* expression was induced by heat stress when fused with the *MhYTP2* promoter but not when fused with the *MhYTP1* promoter (Figure 5A). In contrast, exposure to chilling induced expression when *GUS* was fused with the promoter of either *MhYTP1* or *MhYTP2* (Figure 5A).

After 4 days of either chilling or heat stress, the phenotypes did not differ significantly among any of our transgenic or WT plants (Figure 5B). However, leaf REL values were lower for the transgenics (especially the *MhYTP1-*OE lines) than for the WT in response to chilling, while total chlorophyll concentrations were higher in the OE plants than in the WT under heat stress (Figure 5C). This showed that overexpression of the YTP genes conferred greater resistance to low and high temperatures. Based on the fact that leaves from *MhYTP1-*OE 7 and 15 contained less chlorophyll than did samples from any of the other genotypes under heat stress, *MhYTP2-*OE plants appeared to be more resistant to high temperatures. Therefore, these results indicated that *MhYTP1* has the more dominant role in chilling resistance while *MhYTP2* has a greater role in the heat stress response.

### Overexpression of *MhYTP1* and *MhYTP2* enhances plant resistance to salinity and drought

**Figure 6:**
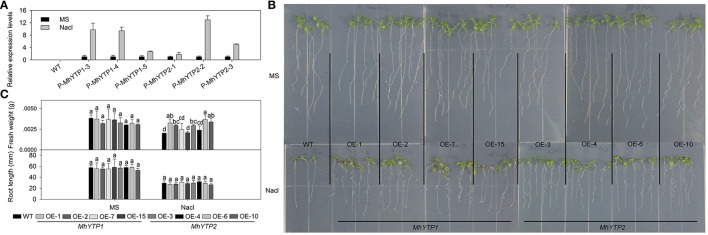
**(A)** Relative expression levels of *GUS* in WT and transgenic plants under normal or high salinity treatment. Data are means ± SD for 5 replicates (at least 20 seedlings each). **(B)** Phenotype comparisons between WT and *MhYTP1-*overexpression plants, and between WT and *MhYTP2*-overexpression plants, in standard MS medium or MS medium with 200 mM NaCl. **(C)** Fresh weights and root lengths of OE and WT *Arabidopsis* plants grown in standard MS medium or MS medium supplemented with 200 mM NaCl. Data are means of 15 replicates ± SD. Values not followed by same letter are significantly different according to Duncan's multiple range test (*P* < 0.05).

**Figure 7:**
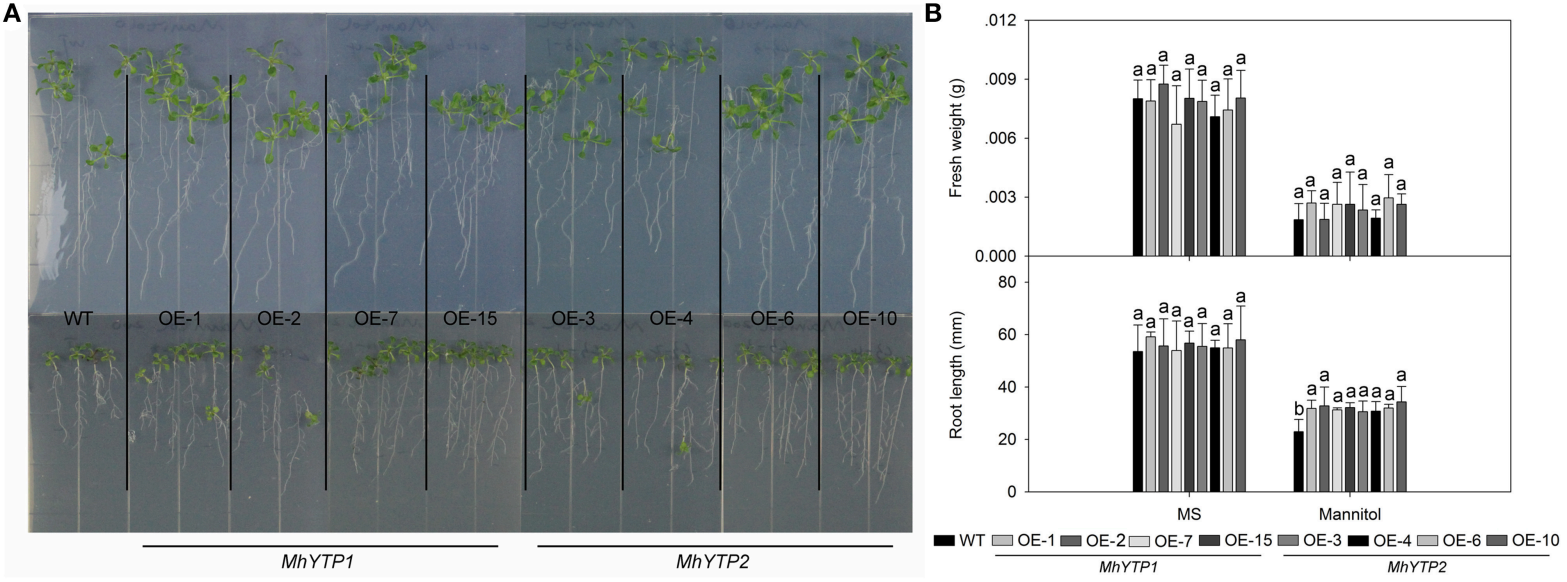
**(A)** Phenotype comparisons between WT and *MhYTP1-*overexpression plants, and between WT and *MhYTP2*-overexpression plants, in standard MS medium or MS medium with 200 mM mannitol. **(B)** Fresh weights and root lengths of OE and WT *Arabidopsis* plants under normal growing conditions or osmotic stress. Data are means of 15 replicates ± SD. Values not followed by same letter are significantly different according to Duncan's multiple range test (*P* < 0.05).

High salinity and drought are severe stresses commonly associated with the cultivation of apple. For high salinity, GUS gene can be increased in plants no matter transferred with *MhYTP1* or *MhYTP2* promoter under NaCl treatments (Figure 6A). Fresh weights were also higher in salinity-stressed OE plants than in similarly treated WT plants, meaning that the former were more resistant (Figures 6B,C). Simulated drought, also termed “physiological drought,” is imposed when plants are treated with high concentrations of mannitol that reduce their capacity to absorb water from the external environment. Although we did not find that mannitol regulated *GUS* expression in our transgenics (data not shown), plants over-expressing either *MhYTP1* or *MhYTP2* were more resistant to mannitol, i.e., drought, and produced longer roots when compared with the WT (Figure 7).

## Discussion

There are limited researches on plant YTPs. Although, MhYTP1 and MhYTP2 could promote leaf senescence and fruit ripening (Wang et al., 2017), their roles in stressed plants have not been confirmed. Each of their promoter regions contains several stress-related *cis*-elements indicating their roles in stress responses. *MhYTP1* and *MhYTP2* are homologous and share more than 90% similarity (Wang et al., 2017), but they differ in their responses to various environmental stimuli.

The TCA-Element occurs in the *MhYTP1* promoter region but not in the *MhYTP2* promoter. However, the latter contains a Box-W1 element (fungal elicitor responsive element). When we compared the responses to exogenous applied SA, expression was reduced when *GUS* was fused to either the *MhYTP1* or *MhYTP2* promoter, and all OE lines proved resistant to treatment with 10 μM SA. This indicated that the two genes are active in SA signaling. Another important participant in the network of plant pathogen defenses is JA. Here, *GUS* expression was induced by treatment with MeJA, especially in plants transformed with the *MhYTP2* promoter. Moreover, gene overexpression plants, particularly the *MhYTP2*-OE lines, are more resistant to MeJA. All of these results led us to conclude that *MhYTP1* plays a more important role in SA signaling and while *MhYTP2* has a greater function in JA signaling.

Three phytohormones—SA, JA, and ethylene (ET)—are crucial to the regulation of signal transduction pathways. However, such control is not achieved through the isolated activation of only a single hormonal pathway but by a complex regulatory network that connects the different pathways. This enables each hormone to either assist or antagonize the others as required to fine-tune the defense response to an individual pathogen. Whereas, the SA-signaling pathway is mainly activated against biotrophic pathogens, the JA-signaling pathway is associated with the development of resistance to necrotrophic pathogens (Glazebrook, 2005; Adie et al., 2007; Verhage et al., 2010). Thus, our findings suggest that *MhYTP1* has a leading role in defenses against biotrophic organisms while *MhYTP2* functions in resistance to necrotrophic pathogens. The presence of an ET-responsive element (ERE) in the *MhYTP2* promoter provides further evidence of a relationship between that gene and hormone signaling, again implying cooperation between JA- and ET-signaling in protecting plants against pathogen infections.

Under various biotic and abiotic stresses, e.g., drought, cold, salinity, and pathogen attacks, ABA signaling regulates the genes responsible for seed germination and stomatal movement (Adie et al., 2007; Beck et al., 2007; Hirayama and Shinozaki, 2007). The promoter regions of *MhYTP1* or *MhYTP2* do not contain any ABA- or drought-related *cis*-elements. Nevertheless, *GUS* expression levels of ABA treated plants transferred with *MhYTP1* promoter were induced. However, the GUS expression levels of ABA treated plants transferred with *MhYTP2* promoter were not significantly different from that of untreated plants. Overexpression plants are more resistant to ABA treatment. Thus, both of the two genes participated in ABA signaling and *MhYTP1* may play a leading role. Then MhYTP1 and MhYTP2 regulated ABA signaling related stress responses.

Almost all promoter regions for *Malus domestica* YTP genes contain at least one anaerobic stress-related *cis*-element (Wang et al., 2014). We found that the promoter region of *MhYTP1* has an ARE while that region of *MhYTP2* have two ARE and a GC-motif. In examining how their expression is regulated when plants are under hypoxia stress due to elevated ROS production, we found that expression of *GUS* fused with either of those promoters increased after MV treatment. This was particularly true for the *MhYTP2* promoter. Likewise, expression of the rat homolog YT521-B is inhibited under oxygen-deficient conditions but is recovered after re-oxygenation (Imai et al., 1998). The first YTP identified in a plant, *AtCPSF30*, also participates in the response to oxidative stress (Chakrabarti and Hunt, 2015). All of these reports suggest that YTPs are functionally homologous in plants and animals. After growing on normal MS or MS media supplemented with 1 μM MV for 7 days, *MhYTP1-*OE and, especially, *MhYTP2-*OE plants performed better that the WT. This difference might have been a result of the *MhYTP2* promoter region carrying more anaerobic stress-related *cis*-elements. Therefore, we can conclude that both genes function in the oxidative stress response, with *MhYTP2* having the dominant role.

The presence of HSEs in the *MhYTP2* promoter region implies that expression of that gene is induced by changes, especially an increase, in temperature. Consistently, the GUS gene fused with the *MhYTP2* promoter was induced by exposure to heat, i.e., 40°C, but not when fused with the *MhYTP1* promoter. In contrast, expression of *GUS*, when fused with the promotor of either gene, was induced by chilling at 4°C. After 4 days of treatment at either temperature, overexpression of these YTP genes conferred resistant to both chilling and heat. This demonstrated that the function of a gene is determined not only by its promoter but also by its structure and that of its protein. As RBPs, the functions of MhYTP1 and MhYTP2 are also determined by their target RNAs. In addition, our transgenic plants also showed greater resistance to high salinity and mannitol (simulated drought) when compared with similarly treated WT plants.

From these results stated above, we can see that *MhYTP1* and *MhYTP2* participate in multiple stress responses. While these sources of stress can be present simultaneously, so also do *MhYTP1* and *MhYTP2* interact with each other, making it difficult to identify individual stress responses. One possible outcome of being exposed to multiple stresses is that plants are better able to develop resistance to more than one type of stress (Bowler and Fluhr, 2000). This phenomenon is called cross-tolerance, which is achieved through a powerful regulatory system that allows plants to adapt quickly to a changing environment (Bowler and Fluhr, 2000; Capiati et al., 2006; Fujita et al., 2006; Suzuki et al., 2012). JA, SA, and ET signaling interact with each other and all play roles in responses to both biotic and abiotic stresses. Miura and Tada (2014) have described the importance of SA in the drought response. This hormone also improves the resistance of *Hordeum vulgare* to water deficits (Bandurska and Stroiński, 2005). The pathogen related (PR) proteins are crucial for resistance against pathogens, and their expression is strongly up-regulated when plants are attacked (Seo et al., 2010). Moreover, many *PR* genes are induced when plants are exposed to abiotic stress (Seo et al., 2010).

We propose that MhYTP1 and MhYTP2 participate in such a variety of stress responses because of their YTH domains. This type of domain can bind with a short, degenerated, single-stranded RNA sequence motif that can be described only by a weight matrix (Zhang et al., 2010). However, an accurate motif sequence has not yet been confirmed. Thus, target RNAs of MhYTP1 and MhYTP2 are numerous, which may be the reason of their wide-spectrum of cellular functions. The way in which RBPs function in stress responses is determined by their promoter *cis*-elements, gene structures, protein structures, and target RNAs. Here, we analyzed the promoter regions of MhYTP1 and MhYTP2 and focused only on how each is induced by stresses. More research involving gene and protein structures, as well as target RNAs, is needed if we aim to improve our understanding of the exact roles of these proteins in stress responses.

In summary, we investigated the responses of *MhYTP1* and *MhYTP2* to various environmental stimuli by studying their promoter *cis*-elements. Although, *MhYTP1* and *MhYTP2* are homologous genes in apple, they still differ in their responses to various treatments. Both genes are active in SA, JA, and ABA signaling and play roles in plant responses to oxidative stress, chilling, heat, salinity, and mannitol-induced physiological drought stress. While *MhYTP1* has the more dominant role in SA and ABA signaling, *MhYTP2* is the main gene involved in JA signaling and oxidative stress responses.

## Author contributions

NW: Experiment design and implementation, manuscript composition; TG, PW, XS, YS, XJ, and BL: Experiment implementation and assistance; XG: Purchase and management of reagents, maintenance of laboratory apparatus; FM: Experiment design and implementation, composition and review of manuscript, financial support for experiments and laboratory apparatus.

### Conflict of interest statement

The authors declare that the research was conducted in the absence of any commercial or financial relationships that could be construed as a potential conflict of interest.

## Supplementary material

Supplementary Table 1 Sequences of primers for vector construction and quantitative real-time PCR.

Supplementary Figure 1 Main stress-related *cis*-elements in *MhYTP1* and *MhYTP2* promoter region. TCA-element, SA signaling-responsive *cis*-element; TC-rich repeat, *cis*-acting element involved in defense and stress responsiveness; ARE, anaerobic induction element; TGA-element, auxin-responsive element; HSE, heat stress response element; ERE, ET-responsive element; Box-W1, fungal elicitor responsive element; GC-motif, anoxic specific induction.

Supplementary Figure 2 Expression levels of GUS gene in *Arabidopsis* transferred with *MhYTP1* or *MhYTP2* promoter regions and WT plants.

Supplementary Figure 3 Expression levels of *MhYTP1* and *MhYTP2* in WT and transgenic *Arabidopsis* plants.

## Abbreviations

ABA: Abscisic acid
CPSF30: cleavage and polyadenylation specificity factor 30
ET: ethylene
GDR: Genome Database for Rosaceae
GUS: β-glucuronidase
JA: jasmonic acid
MeJA: methyl jasmonate
*MhYTP1*: *Malus hupehensis YTP1*
*MhYTP2*: *Malus hupehensis YTP2*
MV: methyl viologen
NCBI: National Center for Biotechnology Information
qRT-PCR: quantitative real-time PCR
RBP: RNA binding protein
REL: relatively electrolyte leakage
SA: salicylic acid
YTH domain: YT521-B homology domain
YTP: YTH domain containing RNA binding protein.
