# Supplementary material for: MhYTP1 and MhYTP2 from Apple Confer Tolerance to Multiple Abiotic Stresses in Arabidopsis thaliana
Source: Front Plant Sci. 2017 Aug 4;8:1367. doi: 10.3389/fpls.2017.01367 (PMC5543281; doi:10.3389/fpls.2017.01367)
Supplement: Supplementary Table 1 — Sequences of primers for vector construction and quantitative real-time PCR. [file Table1.DOCX]

Appendix A Sequences of primers for vector construction and quantitative real-time PCR.

| **Gene** | **Primer** | **Sequence (5**′**–3**′**)** |
| --- | --- | --- |
| **Cloning and vector construction** | | |
| Promoter of *MhYTP1* | P-*MhYTP1*-S | TGCCTTACGACGTCTCAG |
|  | P-*MhYTP1*-A | GTAAGGATACGATCCGCAGG |
| Promoter of *MhYTP2* | P-*MhYTP2*-S | GGGCTTACGATGAAGAGTCGC |
|  | P-*MhYTP2*-A | GTAAGGATACGATCCGCAGG |
| Promoter of *MhYTP1* | 433-P-*MhYTP1*-S | GGGGACAAGTTTGTACAAAAAAGCAGGCTTGCCTTACGACGTCTCAG |
|  | 433-P-*MhYTP1*-A | GGGGACCACTTTGTACAAGAAAGCTGGGTGACAAAGAGTGACACTTGAG |
| Promoter of *MhYTP2* | 433-P-*MhYTP2*-S | GGGGACAAGTTTGTACAAAAAAGCAGGCTGGGCTTACGATGAAGAGTC |
|  | 433-P-*MhYTP2*-A | GGGGACCACTTTGTACAAGAAAGCTGGGTGACAAAGAGTGACTCTTGAG |
| **Quantitative real-time PCR** | | |
| *GUS* | *GUS*-S | GGCTTTGGTCGTCATGAAG |
|  | *GUS*-A | GCGAGGTACGGTAGGAG |
| *Attublin8* | *tublin8*-S | ATAACCGTTTCAAATTCTCTCTCTC |
|  | *tublin8*-A | TGCAAATCGTTCTCTCCTTG |
