# Supplementary figures and images for: MhYTP1 and MhYTP2 from Apple Confer Tolerance to Multiple Abiotic Stresses in Arabidopsis thaliana
Source: Front Plant Sci. 2017 Aug 4;8:1367. doi: 10.3389/fpls.2017.01367 (PMC5543281; doi:10.3389/fpls.2017.01367)

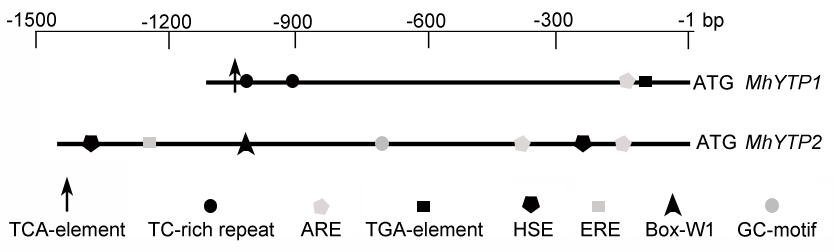

Supplement: Supplementary Figure 1 — Main stress-related cis-elements in MhYTP1 and MhYTP2 promoter region. TCA-element, SA signaling-responsive cis-element; TC-rich repeat, cis-acting element involved in defense and stress responsiveness; ARE, anaerobic induction element; TGA-element, auxin-responsive element; HSE, heat stress response element; ERE, ET-responsive element; Box-W1, fungal elicitor responsive element; GC-motif, anoxic specific induction. [file Image1.TIF]

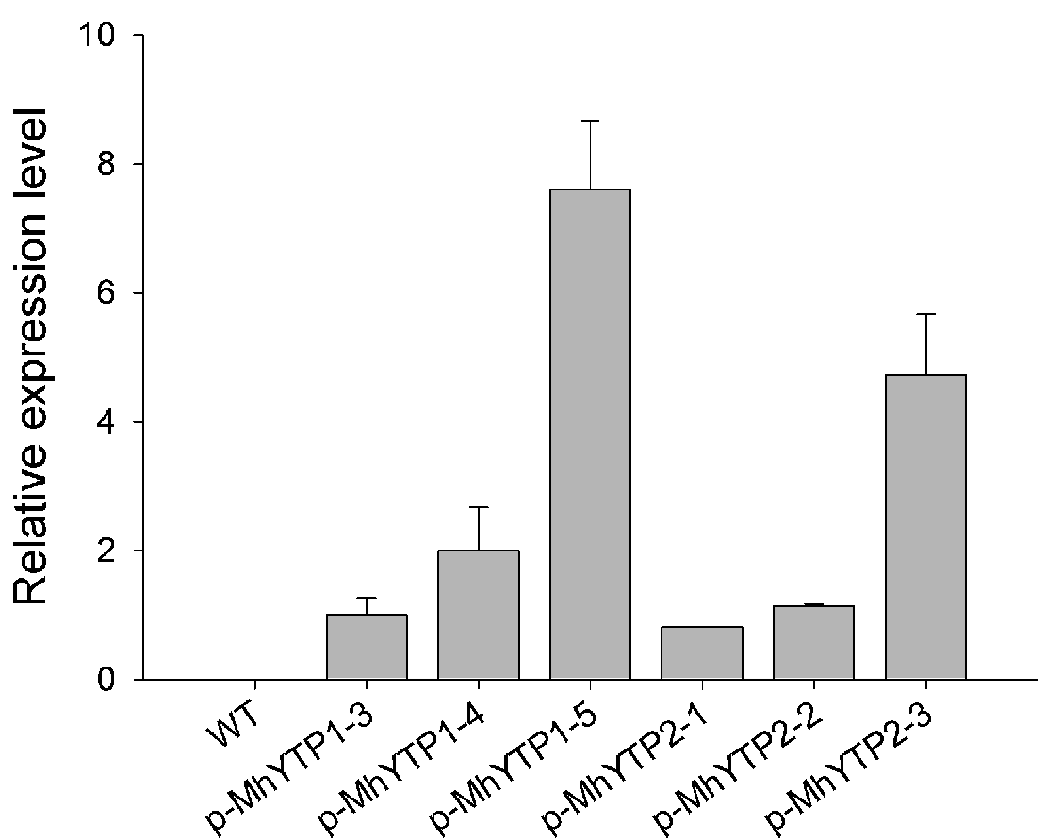

Supplement: Supplementary Figure 2 — Expression levels of GUS gene in Arabidopsis transferred with MhYTP1 or MhYTP2 promoter regions and WT plants. [file Image2.TIF]

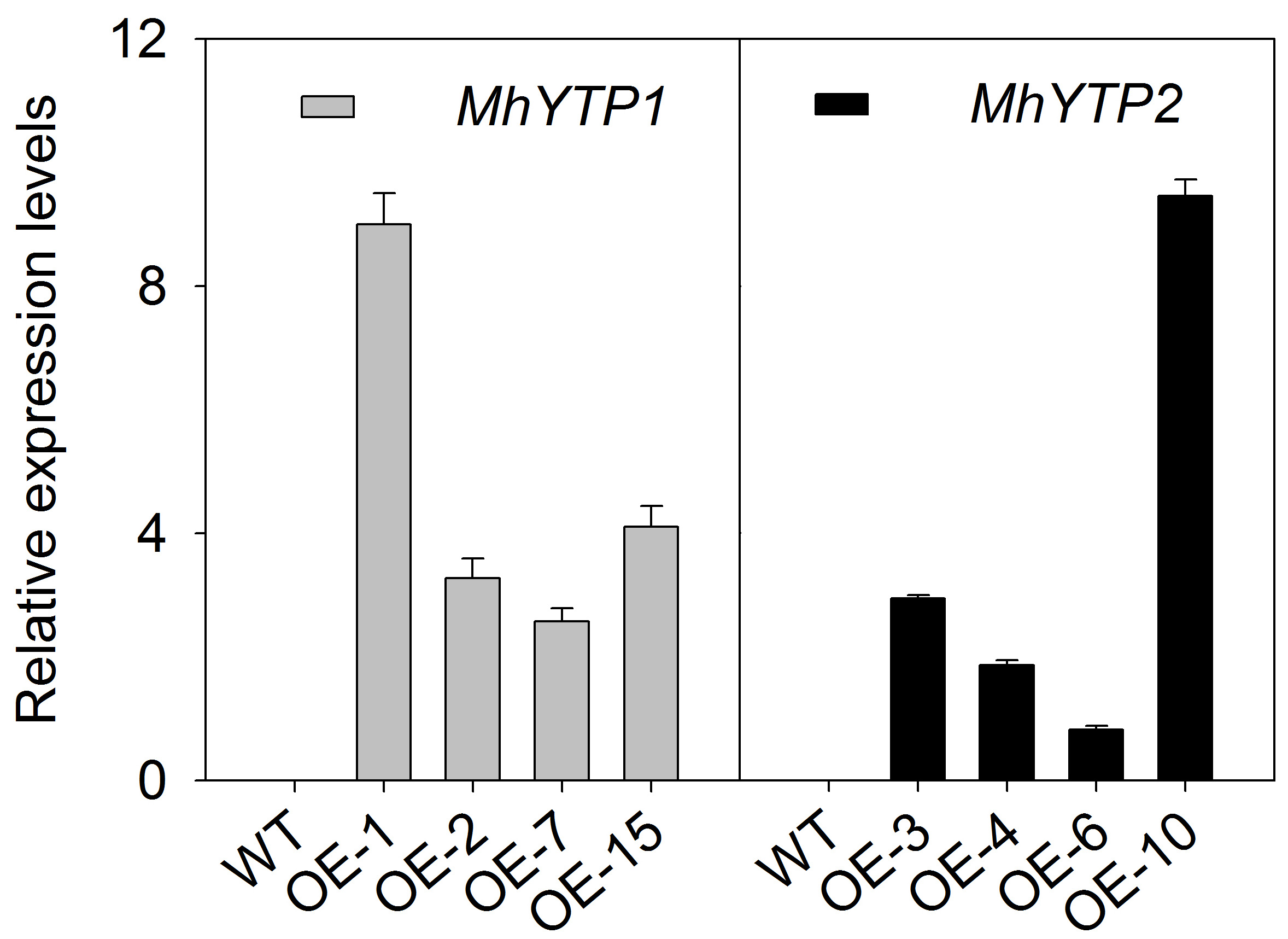

Supplement: Supplementary Figure 3 — Expression levels of MhYTP1 and MhYTP2 in WT and transgenic Arabidopsis plants. [file Image3.JPEG]
